# FDG and FAPI PET–based response evaluation to reduced‐dose nivolumab in pleural mesothelioma: A case report

**DOI:** 10.1111/cpf.70053

**Published:** 2026-02-19

**Authors:** Morten Bentestuen, Anne H. Thostrup, Weronika M. Szejniuk, Zsuzsanna Takacs‐Szabo, Thor L. Rasmussen, Helle D. Zacho

**Affiliations:** ^1^ Department of Nuclear Medicine and Clinical Cancer Research Centre Aalborg University Hospital Aalborg Denmark; ^2^ Department of Clinical Medicine Aalborg University Aalborg Denmark; ^3^ Department of Oncology and Clinical Cancer Research Centre Aalborg University Hospital Aalborg Denmark; ^4^ Department of Radiology Aalborg University Hospital Aalborg Denmark; ^5^ Department of Respiratory Disease Aalborg University Hospital Aalborg Denmark

**Keywords:** CT, FAPI, FDG, PET, pleural mesothelioma, response assessment

## Abstract

Reliable tools for objective response assessment are essential in pleural mesothelioma treated with immunotherapy. We report on a 75‐year‐old man with sarcomatoid pleural mesothelioma who received a reduced nivolumab dose after developing a hypersensitivity reaction during the first infusion. CT, [^18^F]FDG PET/CT and [^68^Ga]Ga‐Fibroblast activation protein inhibitor‐46 (FAPI) PET/CT were performed before and after treatment. Despite the reduced dose, the patient experienced clear clinical benefit, and follow‐up imaging demonstrated substantial morphological and metabolic improvement across all imaging modalities. This case offers an unique opportunity to directly compare response assessment across three complimentary imaging modalities.

## 1 INTRODUCTION

Pleural mesothelioma (PM) is a relatively rare malignancy, most often associated with asbestos exposure. Despite asbestos bans and legislative preventive efforts, the incidence of PM is increasing in certain regions, due to the long latency period of 30–50 years from exposure to development of the disease. Additionally, asbestos use remains prevalent in many low‐income countries (Brims, 2021; Dalsgaard et al., 2019; Popat et al., 2022).

Surgical treatment with pleural decortication is indicated only in carefully selected patients with localized disease, which is estimated to account for fewer than 20% of cases; although, the benefit of surgery compared to chemotherapy alone has recently been questioned following the results of the multicenter phase III MARS 2 trial (Lim et al., 2024; Sørensen et al., 2024). Novel immune checkpoint inhibitors, such as the combination of nivolumab and ipilimumab, have been approved the last couple of years following the positive outcomes of the CheckMate 743 trial (Baas et al., 2021); However, with the introduction of immunotherapy and with new personified oncologic treatment options, reliable tools for objective response assessment are essential to monitor treatment efficacy and avoid unnecessary toxicity.

We present a case of a treatment‐naïve patient with sarcomatoid PM who received a low dose of nivolumab due to hypersensitivity reaction. The patient underwent response assessment using the computed tomography (CT) according to mRECIST 1.1, and two separate Positron Emission Tomography (PET)/CTs with [¹⁸F]fluorodeoxyglucose (FDG) and the novel [⁶⁸Ga]Ga‐fibroblast activation protein inhibitor‐46 (FAPI), analyzed with PET‐based Response Criteria in Solid Tumors (PERCIST) and volumetric response analysis.

This case illustrates a strong treatment response despite markedly reduced nivolumab exposure and provides the first paired FDG/FAPI PET response‐imaging dataset in sarcomatoid PM.

### 1.1 Case presentation

A 75‐year‐old male was referred to chest X‐ray by his general practitioner due to persistent cough over 7 weeks. The chest X‐ray revealed right‐sided pleural effusion with discrete signs of interlobar pleural thickening. The patient underwent thoracocentesis, without cytological confirmation of malignancy.

The contract enhanced CT (ceCT) confirmed diffuse, irregular thickening of the parietal pleura and visceral pleura. PM was suspected, and the patient was referred to a tertiary centre for diagnostic evaluation and treatment. A detailed history revealed additional symptoms, including dyspnoea, fatigue, night sweats, loss of appetite, unintended weight loss, and a recent episode pneumonia. The patient had a 10‐year occupational exposure to asbestos as a bricklayer.

**Figure 1:**
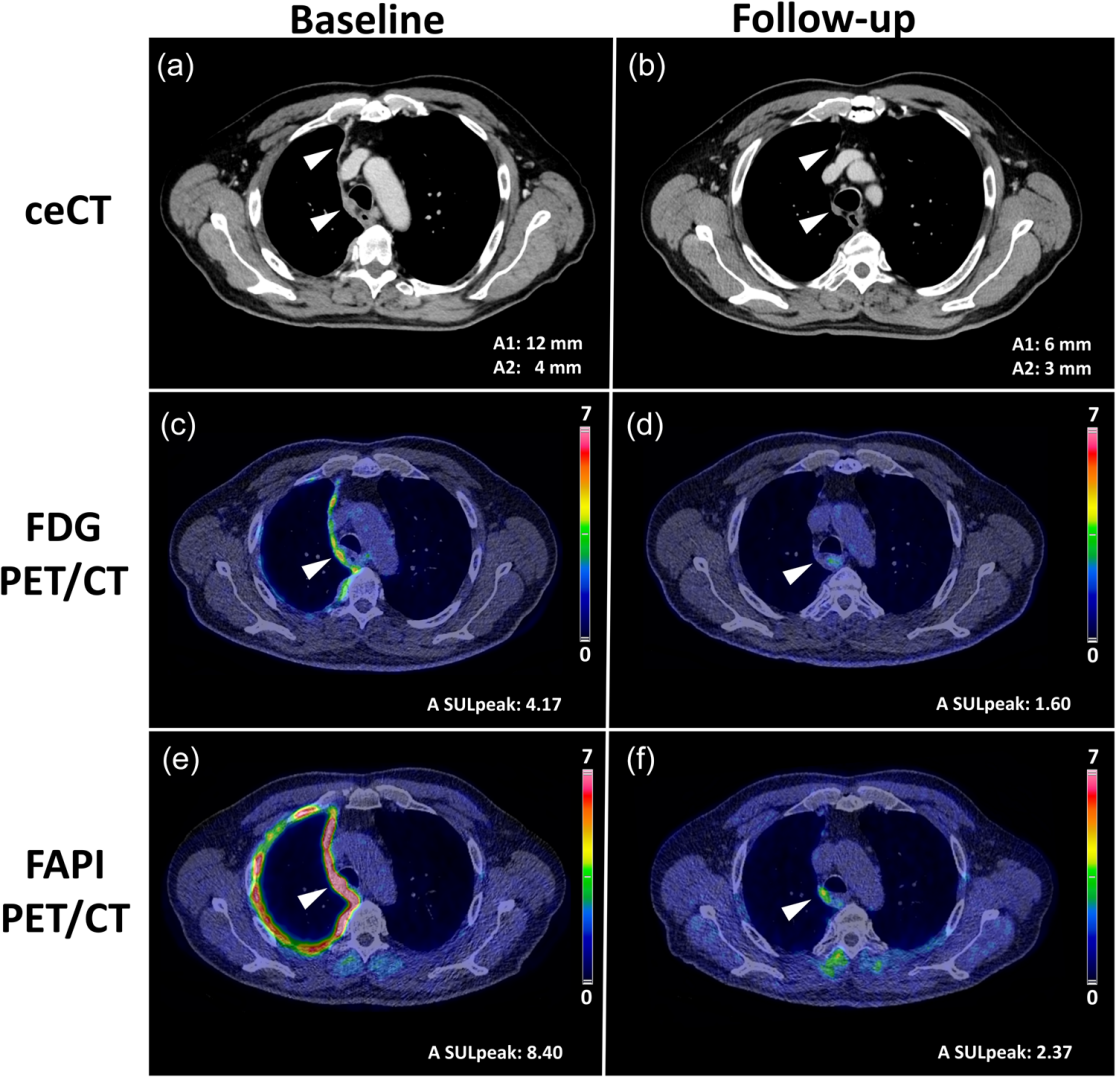
Imaging response assessment of Lesion A, located in the upper medial pleura, with ceCT (a, b), FDG PET/CT (c, d), and FAPI PET/CT (e, f).

As part of the diagnostic workup, the patient underwent FDG PET/CT for staging and biopsy planning. The FDG PET/CT revealed highly elevated FDG‐uptake in the suspected pleural lesions, with a peak standardized uptake value corrected for lean body mass (SULpeak) in the “hottest” lesions of 4.17 (Figure 1C) and with moderate uptake in several thoracic lymph nodes, resulting in a clinical staging T3 N2 M0 (IASLC, 2024). The patient provided informed consent to participate in the clinical trial FAPI‐PM (EUCTIS 2024‐514301‐62‐00, NCT06790082), consisting of additional blinded FAPI PET/CT at primary staging ‐ prior to pleural biopsy procedure, and additional blinded follow‐up FAPI and FDG PET/CTs after 2‐3 cycles of oncologic treatment. FAPI PET at primary staging demonstrated even higher uptake values throughout pleura (hottest lesion SULpeak 8.40, Figure 1E), with no lymph node‐involvement, corresponding to T3 N0 M0. The patient underwent thoracoscopic pleural biopsy of the right lateral pleura, which confirmed sarcomatoid PM; However, the thoracic lymph nodes were not evaluated further. The patient was subsequently referred to first‐line palliative treatment with ipilimumab and nivolumab.

During the first infusion of nivolumab, the procedure was terminated due to a moderate hypersensitivity reaction with sudden intense chest pains, shaking, flushing, inguinal pain, and abrupt blood pressure elevation. Immediate treatment with H₁‐receptor antagonist, morphine, and paracetamol was administered, resulting in resolution of symptoms. Over the following days, the patient experienced self‐limiting fever and intermittent myalgia.

A second treatment attempt 3 weeks later, preceded by pre‐treatment with paracetamol and H₁‐receptor antagonist, was again terminated due to recurrence of hypersensitivity reaction. Further treatment attempts were discontinued, and clinical follow‐up and response ceCT was scheduled 4 weeks later. In total, the patient was estimated to have received 50% of the planned dose of nivolumab and none of the ipilimumab.

**Figure 2:**
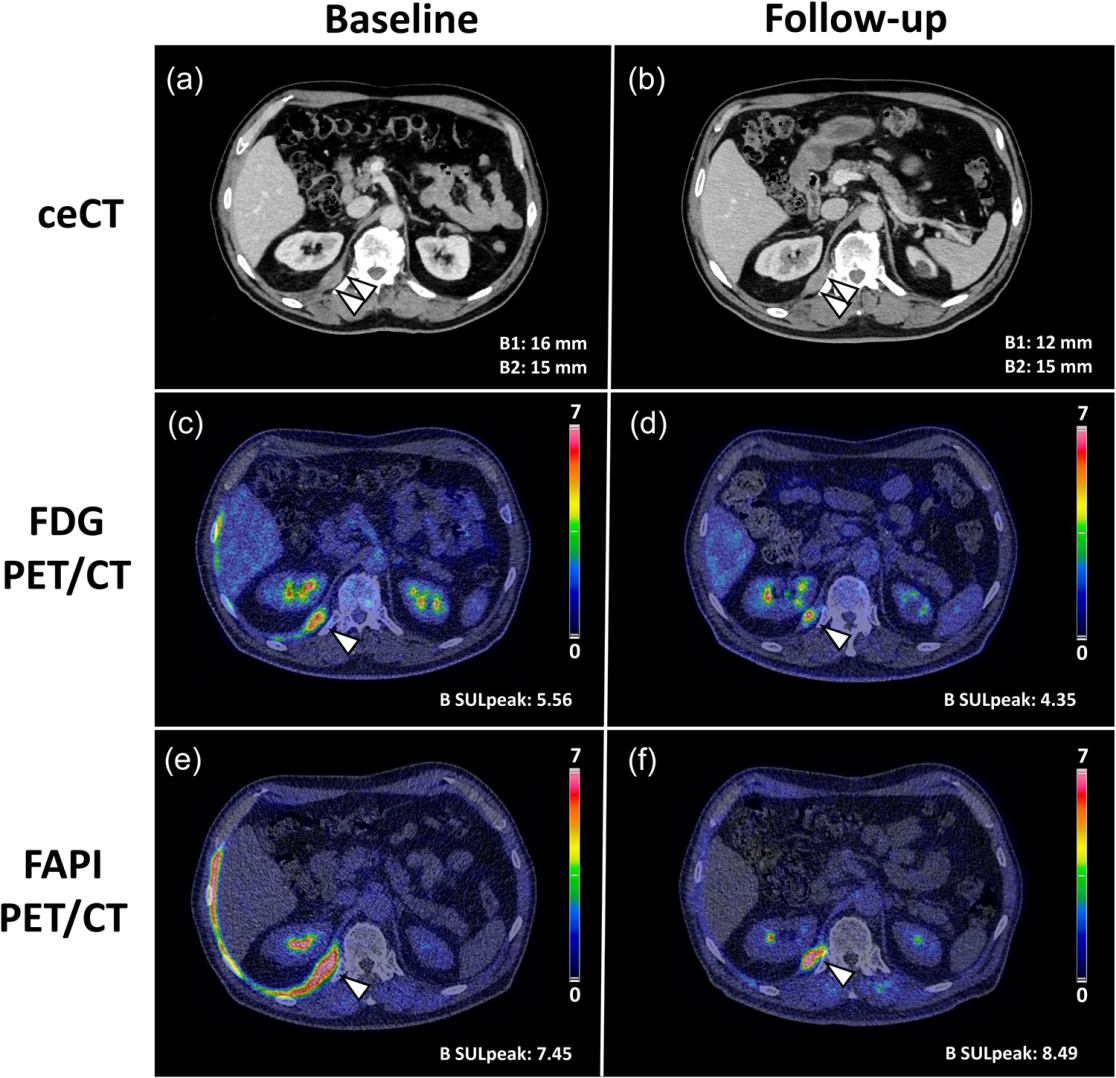
Imaging response assessment of Lesion B, located in the dorsomedial pleura, with ceCT (a, b), FDG PET/CT (c, d), and FAPI PET/CT (e, f).

Overall, follow‐up ceCT showed partial response to treatment according to mRECIST v1.1, with partial response in upper medial pleural lesions (Lesion A, Figure 1a,b), stable disease in lower dorsomedial lesion (Lesion B, Figure 2a,b), and up to 50% reduction in lesion diameters of mediastinal lymph nodes. Summarizing eight target lesions, the mean reduction in diameter was 36%.

PERCIST evaluation of lesion A demonstrated near complete response on FDG PET with a 62% reduction in FDG SULpeak (4.17–1.60; SULmean liver = 1.57) (Figure 1c,d), and a reduction in FAPI SULpeak of 72% (8.40–2.37) (Figure 1e,f). Lesion B, however, demonstrated stable disease, with a 22% reduction in FDG SULpeak (5.56 to 4.35) (Figure 2c,d), and, in contrast, a 14% increase in FAPI SULpeak (7.45–8.49) (Figure 2e,f).

**Figure 3:**
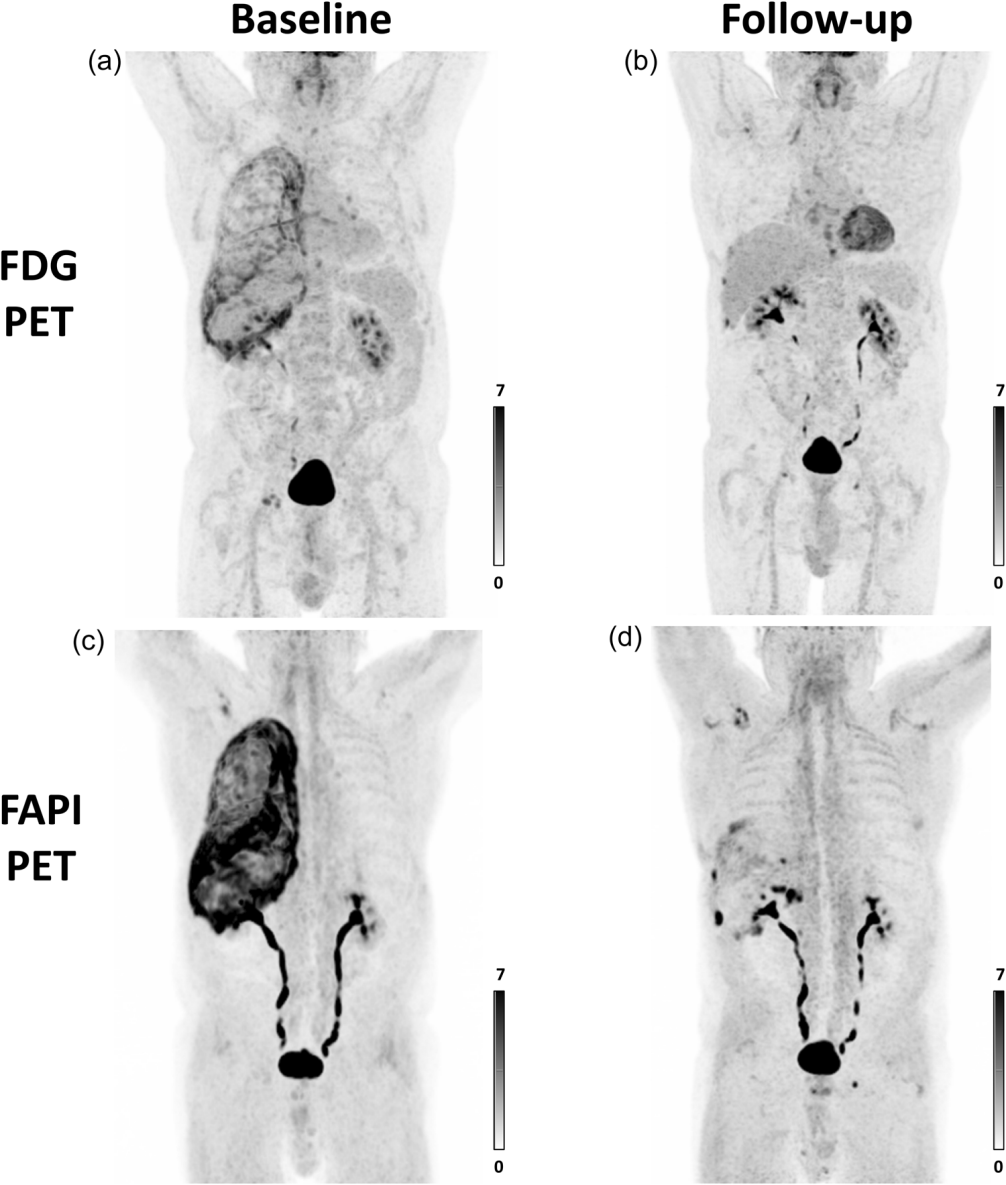
Volumetric response assessment with FDG PET (a, b) and FAPI PET (c, d). On FDG PET there was a 97% reduction in molecular imaging tumour volume (380 to 10 cm^3^) and a 97% reduction in the volume intensity product (1081 to 29 cm^3^ x SUL). On FAPI, there was a 94% reduction in the molecular imaging tumour volume (1195 to 66 cm^3^) and a 95% reduction in the volume intensity product (4605 to 211 cm^3^ × SUL).

Volumetric response assessment, using Syngo.Via lesions scout (Siemens) with a SULpeak cutoff 2.1 (SULmean liver + 2 SD on FDG PET), revealed a 97% and 94% reduction in the molecular imaging tumour volume, and a 97% and 95% reduction in the volume intensity product on FDG PET and FAPI PET, respectively (Figure 3).

On clinical follow‐up approximately 3 months after diagnosis, the patient was in general good health with no limitations in daily activities (performance status 1), had gained 4–5 kg, and reported only mild lateral thoracic pain not requiring analgesics. Continued treatment break and follow‐up was advised.

## 2 DISCUSSION

This case presents some notable highlights. First, the patient had a remarkable response to immunotherapy, even though he only received a minor amount of the planned dosage due to a moderate hypersensitivity reaction to nivolumab. The response was evident both clinically and on all image modalities. Second, both ceCT, FDG, and FAPI PET/CT before and after treatment was available for this patient, resulting in a unique opportunity to compare response assessment with these three imaging modalities. Finally, this is the first case report on response assessment with FAPI PET/CT in PM.

Based on the results of the Checkmate 743 and subsequent trials, international guidelines recommend immunotherapy as first‐line treatment for nonepithelioid PM (Popat et al., 2022). The patient in this case had a moderate hypersensitivity reaction during both the first and second treatment attempts with nivolumab. Though theoretically plausible, an association between immunological adverse reactions and efficacy of immunotherapy has not been established and remains controversial (Postow et al., 2018).

Currently, ceCT is recommended for response assessment of PM. However, this method presents challenges due to the complex growth pattern of PM and is complicated further by the introduction of immunotherapy. To address these issues, the modified RECIST (mRECIST) criteria—specific for PM—and later the immune‐modified RECIST (imRECIST) criteria were developed (Hodi et al., 2018). Response assessment with FDG PET, has not yet been validated. Nevertheless, studies have shown high accuracy in assessing tumour response to both chemo‐ and immunotherapy, as well as in predicting prognosis (Kitajima et al., 2024; Sandach et al., 2022). Furthermore, PET enables response assessment with volumetric parameters, which might be more sensitive compared to PERCIST (Thunold et al., 2025).

In this case, we present the feasibility of FAPI PET for response assessment in PM. FAPI binds to the transmembrane protein “Fibroblast activation protein” (FAP) predominantly expressed by stromal cells of malignant tumours, but in the case of PM, FAP may also be expressed by the cancer cells themselves (Zboralski et al., 2022). However, data on FAPI PET/CT in PM is limited and, to date, only two clinical FAPI PET/CT studies involving a total of 63 patients with PM (five with sarcomatoid subtype) have been published (Güzel et al., 2023; Kessler et al., 2024).

The main limitation of this study is the lack of confirmatory reference standard for treatment response: The overall assessment “partial response” relies on the concordance of findings from the established mRECIST and the FDG PET evaluation, and the clinical follow‐up. For FAPI PET, no standardized criteria exist for response assessment, and the observed changes are of unknown clinical value. The ongoing clinical FAPI PET trial at our department is expected to provide further insights into this unexplored area. In addition, the trial aims to investigate the diagnostic accuracy of FAPI PET/CT compared to FDG PET/CT at the time of diagnosis.

In conclusion, this case report illustrates that even a low dose of nivolumab can show high efficacy in a patient with sarcomatoid PM. Furthermore, it gives illustrative insights to the similarities and differences of the two PET tracers FDG and FAPI—both at baseline and posttreatment, adding to the emerging evidence on FAPI.

## CONFLICT OF INTEREST STATEMENT

The authors declare no conflicts of interest.

## Data Availability

The data that support the findings of this study are available on request from the corresponding author. The data are not publicly available due to privacy or ethical restrictions.
